# Simulation of superselective catheterization for cerebrovascular lesions using a virtual injection software

**DOI:** 10.1186/s42155-021-00242-6

**Published:** 2021-06-14

**Authors:** Sri Hari Sundararajan, Srirajkumar Ranganathan, Vaishnavi Kishore, Raphael Doustaly, Athos Patsalides

**Affiliations:** 1 Department of Neurosurgery, Division of Interventional Neuroradiology, New York Presbyterian Hospital/Weill Cornell Medical Center, 525 East 68th St, New York, NY 10065 USA; 2 Northwestern University Feinberg School of Medicine, 420 East Superior Street, Chicago, IL 60611 USA; 3 GE Healthcare, 500 West Monroe Street, Chicago, IL 60661 USA; 4 Department of Neuro-Interventional Surgery, North Shore University Hospital, Northwell Health, 300 Community Drive, Manhasset, NY 11030 USA

**Keywords:** Cerebrovascular lesion, Endovascular embolization, AVM, dAVF, Mycotic aneurysm, Cone-beam CT, CBCT, Embo ASSIST, Vessel detection software, Virtual catheterization, Virtual injection

## Abstract

**Background:**

This report addresses the feasibility of virtual injection software based on contrast-enhanced cone-beam CTs (CBCTs) in the context of cerebrovascular lesion embolization. Intracranial arteriovenous malformation (AVM), dural arteriovenous fistula (AVF) and mycotic aneurysm embolization cases with CBCTs performed between 2013 and 2020 were retrospectively reviewed. Cerebrovascular lesions were reviewed by 2 neurointerventionalists using a dedicated virtual injection software (EmboASSIST, GE Healthcare; Chicago, IL). Points of Interest (POIs) surrounding the vascular lesions were first identified. The software then automatically displayed POI-associated vascular traces from vessel roots to selected POIs. Vascular segments and reason for POI identification were recorded. Using 2D multiplanar reconstructions from CBCTs, the accuracy of vascular traces was assessed. Clinical utility metrics were recorded on a 3-point Likert scale from 1 (no benefit) to 3 (very beneficial).

**Results:**

Nine cases (7 AVM, 1 AVF, 1 mycotic aneurysm) were reviewed, with 26 POIs selected. Three POIs were in 2nd order segments, 8 POIs in 3rd order segments and 15 POIs in 4th order segments of their respective arteries. The reviewers rated all 26 POI traces – involving a total of 90 vascular segments – as accurate. The average utility score across the 8 questions were 2.7 and 2.8 respectively from each reviewer, acknowledging the software’s potential benefit in cerebrovascular embolization procedural planning.

**Conclusion:**

The operators considered CBCT-based virtual injection software clinically useful and accurate in guiding and planning cerebrovascular lesion embolization in this retrospective review. Future prospective studies in larger cohorts are warranted for validation of this modality.

## Background

Cerebral Arteriovenous Malformations (AVMs), Arteriovenous fistulas (AVFs) and distal aneurysms are dangerous cerebrovascular lesions, potentially leading to intracranial hemorrhage and seizure. They are often managed with endovascular embolization either standalone or in combination with other treatment, such as microsurgery or stereotactic radiosurgery (Ajiboye et al. 2014; Derdeyn et al. 2017; Jiang et al. 2016). Planning cerebrovascular malformation embolization is challenging because it requires a detailed understanding of both the malformation supplying vasculature anatomy and the arterio-venous flow dynamics (Tranvinh et al. 2017). Cone beam CT (CBCT) is a key modality to overcome the intrinsic limitation of the 2-dimensional (2D) projective nature of DSA and enhances 3D visualization of the anatomy (Doerfler et al. 2015; Honarmand et al. 2015).

Leveraging the CBCT, automatic vessel detection software has been demonstrated to facilitate successful embolization in a wide variety of challenging clinical contexts. Though liver embolization was the primary focus for CBCT-based vessel detection software, it’s utility in other anatomic areas have been reported including gastrointestinal arterial bleeds in emergency transarterial embolization (Carrafiello et al. 2016), treatment of renal cell carcinoma, symptomatic benign prostate hypertrophy, small bowel hemorrhage source, and mesenteric pseudoaneurysm (Sundararajan et al. 2019). Safe and effective embolization of challenging lymphatic leakage from the thoracic duct has also been reported when CBCT equipped with guidance software was incorporated (Ierardi et al. 2016). Software integration with add-ons such as metal artifact reduction can further enhance assessment and awareness when planning embolization procedures (Yuki et al. 2016). Use of such a software has been shown to increase embolization success (Cui et al. 2020), with minimal requirements in additional review and processing time, if the staff is properly trained (Carrafiello et al. 2016).

With past success of vessel detection software reducing fluoroscopy time, contrast use, and improving treatment outcomes, use of this technique for endovascular embolization has potential for smooth transition to treat Cerebrovascular Lesions. This study assesses feasibility and clinical utility of virtual injection software used on CBCTs for intracranial endovascular embolization cases.

## Methods

### Patient selection

Our Institutional Review Board approved this retrospective study. Endovascular embolization of AVMs, dural AVFs, or aneurysms performed at our institution between January 2013 and January 2020 were screened, and procedures with CBCTs performed during these procedures were included. Patients with lesions in the proximal vasculature such as saccular aneurysms of the Circle of Willis were excluded from the analysis as navigation is typically not challenging. Procedures that had CBCTs with poor image quality caused by motion were also excluded from this review.

### Image acquisition

All patients underwent endovascular mapping or treatment for intracranial embolization in a biplanar angiographic suite (Innova IGS 630, GE Healthcare, Chicago, IL). CBCTs were acquired during cases in which additional 3-dimensional information about the lesion was required. Selective catheterization under fluoroscopic guidance of the right or left internal carotid or vertebral arteries was performed based on the lesion location. Once the desired point of injection was reached, a DSA or a subtracted fluoroscopic loop was acquired to check catheter position. The CBCT was subsequently acquired using a power-injector to maintain constant injection rates of 4 ml/s during the spin. Three-hundred forty-seven projections were acquired over the 7 s rotation and were transferred to a workstation for 512 × 512 axial reconstructions.

### Virtual injection software

**Fig. 1:**
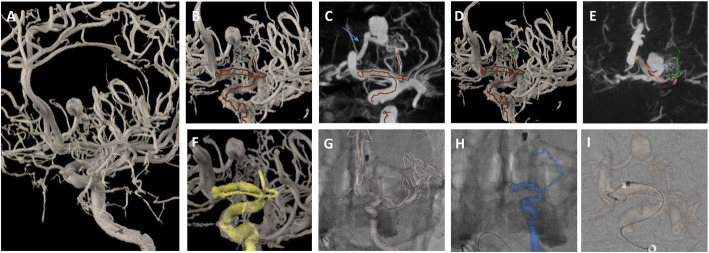
Use of virtual injection software in neurointerventional radiology. Patient with an AVM the left medial frontal lobe with subjacent flow-related aneurysm. The vascular tree was automatically extracted, and the bones removed (**a**). The neuro interventionalist used the virtual microcatheter (the red trace) to review the potential AVM feeders on both the 3D volume rendering (**b**) and the cross-sectional views (**c**). On initial glance, apparent primary feeder to the AVM seems to be the anterior cerebral artery (**c**, blue arrow). However, the software accurately identified this as an en passage vessel supplying the flow-related aneurysm, with subsequent correct identification of the true AVM feeders arising from small caliber lenticulostriate vessels (**c**-**d**, red line). Even though it was also possible to simulate injections from the same points (additional distal contrast flow is simulated past the red line in green, **d**-**e**), the interventionalist were less interested by this feature as the software prediction are unreliable inside the AVM. The simulated catheterizations were saved as 3D model (**f**), which could be used in addition to the full vascular in augmented fluoroscopy (**g**- full vascular tree; **h**- targeted vessel; **i** – example of catheterization guidance)

Virtual injection software (Embo ASSIST, GE Healthcare; Chicago, IL) has been developed to simulate selective catheterization from a proximal CBCT (Soliman et al. 2019). After semi-automatic extraction of the vascular tree (Fig. 1a), the user can dynamically simulate any distal catheterization of the vasculature using the mouse pointer as a virtual microcatheter tip. As the pointer is used to select a location in the vasculature, a virtual trace of the vessel proximal to the pointer is instantly created in red (Fig. 1b-c). It represents the virtual catheter navigation from the point of injection to the location selected via the mouse pointer. At the same time, all vessels distal to the pointer are highlighted in green (Fig. 1d-e), mimicking an injection from this point both on the volume rendered vascular tree and on cross-sectional views.

### Software performance and clinical utility evaluation

All included patients’ studies were transferred to a workstation (Advantage Windows, GE Healthcare, Chicago, IL) for analysis. Two neuro interventionalists, with 15 years (Reviewer A) and 5 years (Reviewer B) respective experience processed independently the CBCT using the virtual injection software. Semi-automatic extraction of the vascular tree was performed and adjusted by the user if needed. Virtual Injection (VI) feature was then used to simulate different treatment scenarios.

Each neuro-interventionalist identified Points of Interest (POIs) using virtual catheterization on the volume rendered vascular tree. A POI was defined by its segment location on a specific artery. Thereby, POIs selected by the different reviewers on the same segment from the same artery were considered equivalent, even if their 3-dimensional locations were slightly different. Conventional vessel naming was used in denoting POI location with rising order from 1st to 4th order denoting rising levels of vessel distality. For each reviewer, selected POIs were characterized by their clinical purpose for identification and segmental location. POIs whose proximal vessels exited the CBCT field of view were excluded.

The virtual trace associated with each selected POI was analyzed on the CBCT cross-sectional views to assess trace accuracy for each vascular segment, from the most proximal one to the most distal one (Fig. 1e). The accuracy of each trace was ranked per segment following a 4-points scale described in Table 1.

**Table 1:** Accuracy Classifications for Traced Vessels

Classification	Definition
Accurate	Vessel tracking is correct from the vascular tree root to the POI
Clinically Usable	Vessel tracking is correct for major branch points but incorrectly traces distal vessels
Not Clinically Usable	Vessel tracking is wrong for major branch points and distal vessels
Not Detected	Vessel cannot be tracked using VI

The trace was then exported as a separate 3D model of the vessel that stretched from the vascular tree root to the chosen POI. This 3D model was highlighted on the vascular tree in the 3D volume rendering and could be used as augmented fluoroscopy to guide the procedure during a live case (Fig. 1f-i).

A series of clinical utility questions were asked after the review of each case to gauge the potential benefits of virtual catheterization feature on a 3-points Likert scale from 1 – no benefit to 3 – very beneficial. Utility questions are displayed in Table 2.

**Table 2:** Clinical Utility Questions

How helpful was VI in distinguishing between close arterial branches?	
How helpful would you expect VI to be in decreasing necessary catheterizations?	
How helpful would you expect the VI 3D overlay to be in navigating to the POI?	
How helpful would you expect VI to be in decreasing contrast usage?	
How helpful would you expect VI to be in decreasing the necessity of 2D roadmapping during procedures?	
How helpful would you expect VI to be in improving your ability to select the correct vessels for embolization?	
How helpful would you expect VI to be planning a procedure?	
Were you able to select (an) embolization point(s) for this case using VI?	

### Statistical analysis

Analyses were conducted using Microsoft® Excel® (Build 11,929.20708), and descriptive statistics were used to represent the data. Inter-reader agreement percentage on clinical utility was measured per question and describes the percentage of scores on which both reviewers agreed.

## Results

### Patients

Nine patients with cerebrovascular lesions involving distal vasculature had CBCTs performed during the endovascular mapping or treatment for intracranial embolization. CBCTs were performed primarily when the operator felt the utility of added cross-sectional vessel imaging would be of benefit in understanding the pathologic vascular anatomy in question. A total of 7 AVM, 1 AVF, and 1 mycotic aneurysm procedures were processed using the virtual injection software (Table 3). No CBCT was excluded due to poor image quality.

**Table 3:** Summary Characteristics

**Variable**			
**CBCTs (n)**		9	
Injection Point	Right Internal Carotid Artery	4	
	Left Internal Carotid Artery	2	
	Right Vertebral Artery	1	
	Left Vertebral Artery	2	
		**Reviewer A**	**Reviewer B**
**POIs (n)**		13	21
Avg Segmental Location		3.3	3.6
Total Segments		43	76
Segmental Location	1st order	–	–
	2nd order	2	1
	3rd order	5	6
	4th order	6	14
Arterial Location	Posterior Cerebral Artery	5	5
	Middle Cerebral Artery	2	5
	Anterior Cerebral Artery	2	6
	Superior Cerebellar Artery	–	2
	Anterior Choroidal Artery	1	1
	Posterior Inferior Cerebellar Artery	–	1
	Inferolateral Trunk	–	1
	Lenticulostriate Artery	1	–
	Posterior Communicating Artery	1	–
	Meningohypophyseal Trunk	1	–
Purpose of Selection	Embolization	13	16
	Eliminate Suspect Feeder	–	4
	Confirm En Passage	–	1

### Virtual injection software performance

**Fig. 2:**
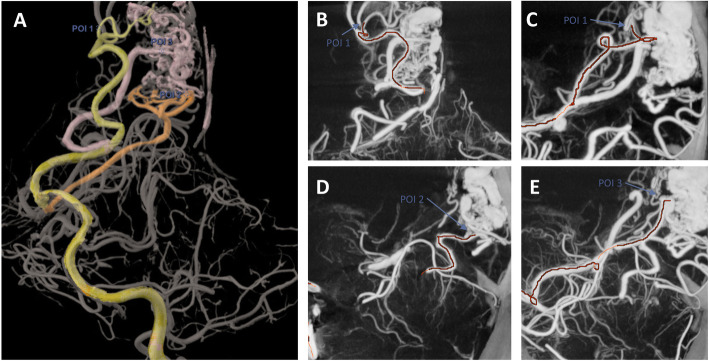
Retrospective review of an AVM. Each potential AVM supplying vessel was interrogated using virtual catheterization software on the 3D volume rendering. Three Points Of Interest (POIs) were identified and exported as 3D models (**a**). The virtual catherization trace was then reviewed for each POI on cross sectional views to assess its accuracy, as shown in coronal and sagittal reconstruction for POI 1 (**b-c**), and sagittal reconstructions for POI 2 (**b**) and POI 3 (**c**)

**Fig. 3:**
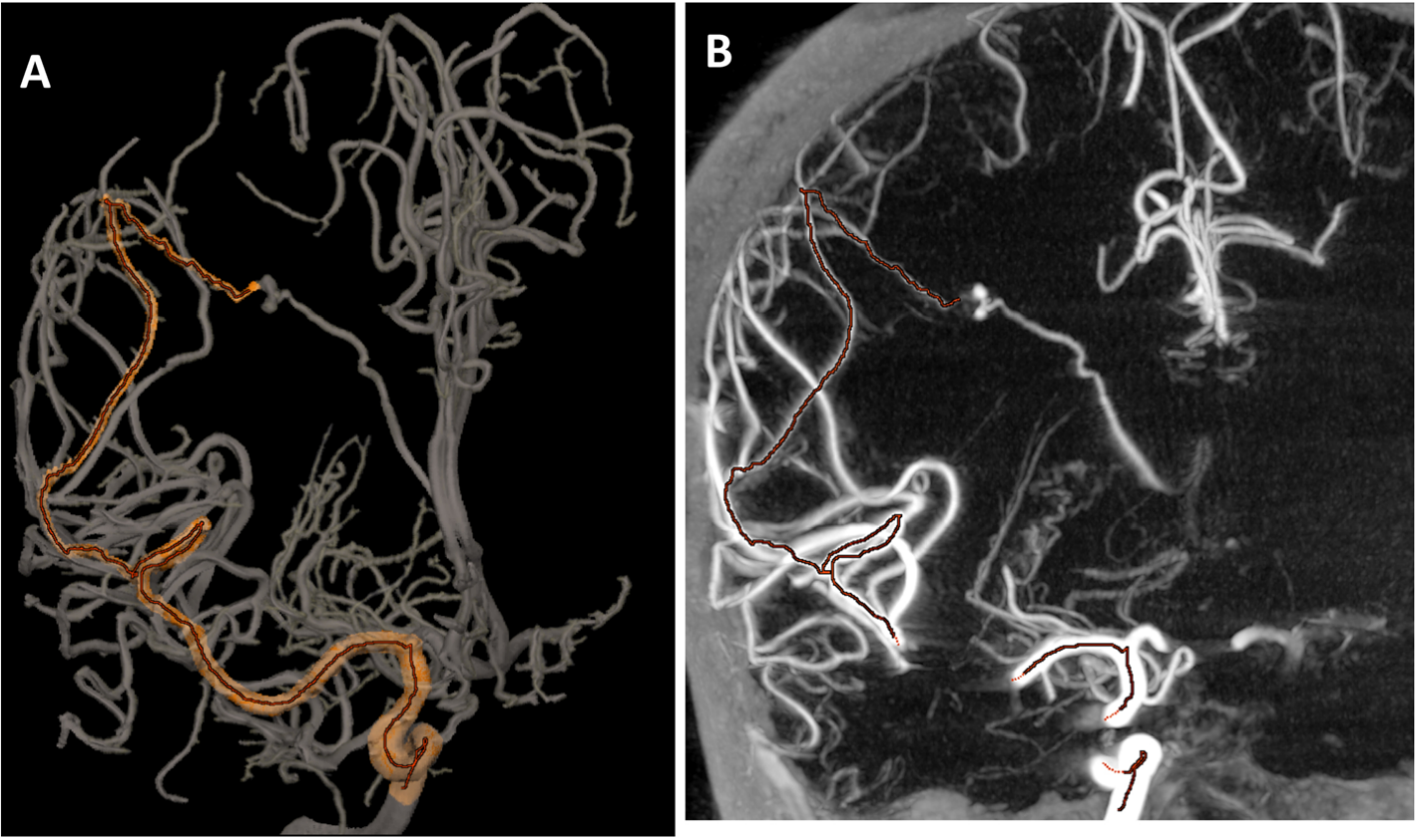
Retrospective review of a distal aneurysm. Virtual catheterization software was also evaluated for distal embolization, such as in a context of mycotic aneurysm. The software was used to create a 3D roadmap based on the CBCT (**a**). The accuracy of the 3D roadmap was assessed using the virtual catheterization trace on the cross-sectional views (**b**)

Twenty-six independent POIs were selected for review by the neurointerventionalists: 3 POIs of 2nd order, 8 POIs of 3rd order and 15 POIs of 4th order in their respective arteries. Eight of the 26 POIs were selected by both reviewers across 6 cases for the purpose of embolization or elimination of a suspect feeder. Reviewer A identified 5 other independent POIs for the purpose of embolization. Reviewer B identified 13 other POIs either for embolization (Sundararajan et al. 2019), to confirm an en passage vessel (Ajiboye et al. 2014), or to eliminate a suspect AVM feeder vessel (Tranvinh et al. 2017). Twenty-six traces to POIs, involving 90 vascular segments were analyzed by the reviewers, as shown in the examples in Figs. 1, 2, and 3. All of them were rated as accurate, meaning that the vessel tracking was estimated correct from the vascular tree root to the POI for reviewers.

### Clinical utility

The average utility score across the 8 questions was 2.7 out of 3 for Reviewer A and 2.8 for Reviewer B, showing that both reviewers retrospectively evaluated Virtual Injection as clinically useful for cases of intracranial embolization.

The Virtual Injection tool was rated most useful for procedure planning and guidance (Table 4). The use of Virtual Injection in distinguishing between close arterial branches, in navigation to the POI, planning procedures and identifying embolization points was rated as very beneficial, with an average score above 2.8 (Inter-reader agreement of 78%, 55% and 66% respectively). This technology was also rated as valuable to select an embolization point, to decrease the amount of contrast used, the necessity of 2D roadmapping and the number of catheterizations, with an average score from 2.6 to 2.7 (Inter-reader agreement of 66% for the 3 questions). The largest difference between the two reviewers’ ratings was regarding the utility of the software in navigation to the POI as a 3D overlay. Reviewer A’s average rating was a 2.6 and Reviewer B’s average rating was a 3. Table 4 shows the average clinical utility scores and inter-reader agreement for the cases.

**Table 4:** Clinical Utility Questions and Scores

Question	Average Utility Score (Reviewer A Avg, Reviewer B Avg)	Inter-Reader Agreement Percentage
Distinguishing between close arterial branches	2.9 (2.8, 3)	77.8%
Decreasing necessary catheterizations	2.6 (2.6, 2.6)	66.7%
Navigation to POI as a 3D overlay	2.8 (2.6, 3)	55.6%
Decreasing contrast usage	2.6 (2.7, 2.6)	66.7%
Decreasing 2D roadmapping during procedures	2.6 (2.7, 2.6)	66.7%
Improving ability to select vessels for embolization	2.7 (2.7, 2.8)	66.7%
Planning a procedure	2.8 (2.8, 2.8)	66.7%
Identification of embolization point(s)	2.8 (2.8, 2. 8)	66.7%

## Discussion

The development of new devices and embolic agents has expanded the endovascular approach for cerebrovascular malformation treatment (Pierot et al. 2013), whether in preparation for microsurgery (Natarajan et al. 2008), radiosurgery (Blackburn et al. 2011), or occasionally standalone. The interventionalist must know several characteristics of the malformation in order for the highest chance of successful embolization: the exact vascular supply of the malformation, the number of feeders and feeding pedicles, the flow dynamics of the feeders, and the characteristics of draining vessels or associated aneurysms. Obtaining a clear and exact picture of these characteristics is difficult with especially dense, complex, and very distal lesions, rendering planning and subsequent embolization much more technically challenging.

Any number of feeder arteries may supply a given lesion, comprising two main patterns of flow: the supplying artery either projects a variable number of feeder arteries to the lesion (while the main artery supplies brain tissue past the branch point) or directly supplies the lesion. The former vascular pattern, known as vessel in passage feeders, levies a higher risk of neurological deficits after treatment as a result of the risk of embolization or trauma to the main vessel. It is thus is very helpful to be able to visualize single branches of malformations with multiple feeders, elucidating how exactly the malformation receives flow from feeders. When embolizing malformations, it is important to first embolize pedicles that are the largest and/or feed distal veins and aneurysms. Characteristics of the lesion itself also play a crucial role in dictating embolization approach. For instance, more superficial malformations derive their supply most commonly from peripheral branches of the anterior, middle, and/or posterior cerebral arteries, while larger and deeper malformations may derive their supply from lenticulostriates, choroidals, and similarly located central arterial vessels. The deeper the lesion, the more complex and technically difficult the embolization can potentially be due to overlapping of various vasculature on angiographic imaging alone (Crimmins et al. 2015).

Given the need to exactly ascertain these characteristics prior to embolization, the use of advanced imaging technique and CBCT during embolization of cerebrovascular lesions has exploded in recent years. Lin et al. presented time-resolved CBCT as a technology providing both anatomic and hemodynamic evaluations for real-time classification of AVMs (Lin et al. 2018), and Sandovia-Garcia et al. suggested that this technique could replace conventional CBCT and multiple 2D DSA in diagnostic and therapeutic procedures (Sandoval-Garcia et al. 2017). In their study, Blanc et al. have demonstrated the applicability of CBCT-based 3D-Roadmap for superselective catheterization during AVM embolization (Blanc et al. 2015).

To the best of our knowledge, this retrospective study is the first one to assess a newly released virtual injection software in a context of cerebrovascular lesion diagnosis or treatment. Compared to the other advanced visualization techniques listed above, virtual injection software is designed to minimize CBCT review time and microcatheter manipulations, by simulating selective catheterizations. All superselective catheterizations simulations in this study were evaluated as accurate, even for the more distal segments. This preliminary result suggests that the application of the virtual catherization technology is safe in cerebral CBCTs.

There were minor differences between Reviewer A (15 years of experience) and Reviewer B (5 years of experience). Selective catheterizations simulations seem more useful for less experienced operators, as illustrated by the increased number of POIs selected by Reviewer B and his higher average utility score compared to Reviewer A. Overall however, both neurointerventionalists described virtual catheterization technology as very useful. Potential benefits were identified in procedure planning: identification of the supplying branches, embolization points and minimizing microcatheter manipulations. These observations corroborate the results published for other anatomies, such as trans-arterial liver-directed therapies, where similar technologies have been available for years (Cui et al. 2020). The software was also evaluated as useful for procedure guidance and to segment the targeted vessels in preparation of an overlay to be used with live fluoroscopy. A few studies reported the benefit of augmented fluoroscopy for neurointerventional procedures in increasing operator confidence, reducing radiation dose, and reducing contrast injected into the patient. In these publications, volumes used for 3D-roadmapping were mainly extracted from pre-op imaging (Zhang et al. 2017; Ruijters et al. 2011; Zhang et al. 2016; Kishore et al. 2020), with no time constraint to segment the structures of interest, but inherent risks of misregistration due to change in patient position. In some other studies, the whole vasculature from the CBCT was used as overlay (Blanc et al. 2015; Jang et al. 2016), as careful segmentation of the targeted vessels was too time-consuming to be performed intraoperatively. In this scenario, the interest in using augmented fluoroscopy might be limited due to many vessels overlapping on the 3D roadmap and causing cluttered visualization.

The software has certain limitations. The software is FDA approved for utilization only with angiography and cone-beam CT data acquired on GE hardware. However, the software can be utilized on any single plane and bi-plane GE angiography suite regardless of its age or generation as long as it is a device with cone-beam CT acquisition capability. An artificially wrong course can potentially be mapped if there is significant venous contamination during the cone-beam CT as subjacent opacified venous structures would interfere with the software’s ability to detect clear arterial trajectories. This can also been seen if there is broad or excessive arteriovenous shunting proximal to the malformation’s nidus. As such, the software’s utilization is best exemplified in clinical situations in which navigation towards a distal aneurysm or focal AVM nidus (and potentially an arteriovenous fistula communication point) without excessive surrounding venous contamination proximal to the target is needed.

This study had notable limitations. The retrospective design did not allow us to assess the software performance in real time procedure settings. The small number of patients included is another concern. The small field of view is an intrinsic limitation of such CBCT based vessel detection software, as the software does not track all vessels exiting the CBCT volume. Another common reason given by the reviewers for low utility scores is some patients’ straightforward anatomy, where a quick review of CBCT alone was enough to complete the procedure effectively and efficiently. Additionally, clinical judgment is warranted to select the utility of performing CBCT in a given cerebrovascular malformation, as each patient’s respective anomaly may possess varying degrees of vessel friability and highly suspicious features (such as flow-rated or intra-nidal aneurysms) making them prone to vessel injury ranging from spasm to potential rupture. Such risks may be mitigated with alterations in acquisition technique. These include allowing a rise to achieving final rate of contrast injection, injecting from a common carotid artery rather than direct internal carotid, thereby distributing the total volume of contrast across other surrounding vascular territories (though this may degrade the ability of the software to identify target vessels in the setting of more surrounding opacified vessels).

We believe virtual injection software will promote the adoption of augmented fluoroscopy by enabling rapid segmentation of the targeted vessels. Specifically, this technology adds value via its capability of identifying the appropriate vascular course on 3D images with subsequent allowance for use of the selected route to navigate more efficiently during real-time catheterization and angiography. This software had the potential to be useful not only in the navigation to distal aneurysms and AVM niduses. Precise target vessel selection during catheter directed delivery of chemotherapeutic or immunomodulating agents to intracranial neoplasia is one possible utility of this software, noting that the added reassurance of accurate vessel supply to a given lesion has been of great clinical benefit in the interventional oncology realm of non-neural axis viscera (Cui et al. 2020). Similarly, navigation to branches of the external carotid artery during embolization of head and neck region neoplasia, vascular malformations, or arteriovenous fistula feeders is also technically feasible, noting software limitations that may arise as described earlier.

## Conclusion

In conclusion, virtual catheterization technology simulates accurately selective catheterization in cerebral CBCT. It was considered clinically useful in guiding and planning cerebrovascular lesion embolization by the operators in this retrospective review. As such, this software can potentially be useful for operators with less experience in navigating to distal targets with several possible vascular trajectories on conventional angiography. Future prospective studies in larger cohort are warranted for validation of this modality.

## Data Availability

The datasets used and/or analyzed during the current study are available from the corresponding author on reasonable request.

## Abbreviations

2D: 2 Dimensional
3D: 3 Dimensional
AVM: Arteriovenous Malformation
AVF: Arteriovenous Fistula
CBCT: Cone Beam Computed Tomography
POI: Point of Interest
